# Effects of *Trichinella spiralis* excretory-secretory antigens on expression of indoleamine 2, 3-dioxygenase on dendritic cells *in vitro*

**DOI:** 10.1051/parasite/2025018

**Published:** 2025-04-16

**Authors:** Wenhao Yu, Xuhong Yuan, Peng Zhai, Xiaoyun Li, Caixia Han

**Affiliations:** College of Veterinary Medicine, Northeast Agricultural University Harbin China

**Keywords:** *Trichinella spiralis*, Indoleamine 2, 3-dioxygenase, Dendritic cells, Immune tolerance, siRNA interference

## Abstract

Indoleamine 2, 3-dioxygenase (IDO) is a potent immunoenzyme found in dendritic cells (DCs). Research has demonstrated that *Trichinella spiralis* induces IDO expression in the host immune response through its excretory-secretory (ES) antigens. However, the role of IDO in the immune response to *T. spiralis* remains unclear. To examine the effects of *T. spiralis* ES antigens on IDO expression in DCs *in vitro*, assessments were conducted using qRT-PCR, Western blotting (WB), flow cytometry, and siRNA transfer. The findings indicated that ES antigen stimulation upregulated IDO expression in DCs *in vitro*. Furthermore, ES antigen significantly enhanced the expression of the proinflammatory cytokines TNF-α and IFN-γ, along with the anti-inflammatory cytokine IL-10, downstream of IDO in DCs. Flow cytometry analysis confirmed that surface molecules CD40, MHC-II, CD80, and CD86 on DCs were upregulated following stimulation with ES antigen and lipopolysaccharide (LPS). Compared to the ES antigen alone, siRNA620 effectively inhibited IDO levels, demonstrating a statistically significant reduction. Continuous stimulation of DCs by ES antigens may lead to immune tolerance through the activation of IDO-mediated inflammation-associated factors. These results suggest that IDO expression in DCs plays a crucial role in *T. spiralis* infection.

## Abstract (fr)

L’indoleamine 2,3-dioxygénase (IDO) est une immunoenzyme puissante présente dans les cellules dendritiques (CD). Des recherches ont démontré que *Trichinella spiralis* induit l’expression d’IDO dans la réponse immunitaire de l’hôte via ses antigènes excrétés-sécrétés (ES). Cependant, le rôle de l’IDO dans la réponse immunitaire à *T. spiralis* reste incertain. Afin d’examiner les effets des antigènes ES de *T. spiralis* sur l’expression d’IDO dans les CD *in vitro*, des évaluations ont été réalisées par qRT-PCR, Western blot (WB), cytométrie de flux et transfert d’ARNsi. Les résultats ont indiqué que la stimulation par les antigènes ES régulait positivement l’expression d’IDO dans les CD *in vitro*. De plus, les antigènes ES ont significativement augmenté l’expression des cytokines pro-inflammatoires TNF-α et IFN-γ, ainsi que de la cytokine anti-inflammatoire IL-10, en aval de l’IDO dans les DC. L’analyse par cytométrie de flux a confirmé que les molécules de surface CD40, MHC-II, CD80 et CD86 sur les DC étaient régulées positivement après stimulation par les antigènes ES et le lipopolysaccharide (LPS). Comparé aux antigènes ES seul, le siRNA620 a inhibé efficacement les taux d’IDO, démontrant une réduction statistiquement significative. La stimulation continue des DC par les antigènes ES pourrait induire une tolérance immunitaire via l’activation de facteurs associés à l’inflammation médiés par l’IDO. Ces résultats suggèrent que l’expression d’IDO dans les DC joue un rôle crucial dans l’infection à *T. spiralis*.

## Introduction

Trichinellosis is a globally prevalent foodborne zoonotic disease caused by the ingestion of raw or inadequately cooked meat containing infectious *Trichinella spiralis* larvae [18]. Infection with *T. spiralis* poses a significant challenge to host immunity, as the parasite can complete its entire life cycle within a single host [35]. To achieve this, *T. spiralis* modifies environmental conditions that support the survival of both the host and the parasite [12, 42]. The parasite develops in humans by employing various mechanisms to evade the host immune response. However, the precise mechanisms underlying immune evasion remain unclear and require further investigation.

Based on different developmental stages, *T. spiralis* antigens are categorized into worm antigens, surface antigens (SA antigens), and excretory-secretory (ES) antigens. ES antigens activate the nucleotide-binding oligomerization domain-containing protein 1 (NOD1) receptor pathway in mouse peritoneal macrophages, regulating nuclear factor kappa-B (NF-κB) expression and other key downstream factors. Additionally, ES antigens can induce immune tolerance in macrophages for a limited duration [44]. During *T. spiralis* infection, ES antigens directly enter tissue fluid, lymph, and blood, participating in the host immune response and serving as key target antigens that elicit specific immune responses [33]. These antigens influence the host immune response through antigen-presenting cells (APCs), inducing APCs to activate T helper 2 (Th2) cells and anti-inflammatory responses, while promoting regulatory T-cell activation both *in vitro* and *in vivo* [17].

*Trichinella spiralis* and its immunoregulatory molecules interact with various immune cells, modulating cytokine expression and regulating the host immune system. The immunoregulatory process involves antigen processing and presentation, lymphocyte activation and proliferation, and cytokine release [3]. Infection with *T. spiralis* enhances the expression of interleukin-4 (IL-4) and interleukin-10 (IL-10), while reducing interferon gamma (IFN-γ) and interleukin-17 (IL-17) levels. The upregulation of Th2 cytokines, particularly IL-4, suppresses the T helper 1 (Th1) cell-mediated immune response against foreign antigens, which may have beneficial effects in autoimmune conditions. Furthermore, *T. spiralis* induces the Th2 phenotype and promotes cytokine production, including IL-4, IL-5, and IL-13, which are essential for the establishment and maintenance of the Th2 response [1].

The immune system maintains equilibrium between immunity and tolerance to defend the host against infections, while minimizing local tissue damage [24]. Dendritic cells (DCs), like APCs, play a critical role in immune resistance. These cells activate primary T-cells and coordinate immune responses, contributing significantly to immune regulation [20, 30] and T-cell tolerance induction [25]. Indoleamine 2,3-dioxygenase (IDO), an enzyme produced by DCs, facilitates the establishment and maintenance of immunological tolerance [26]. Although several hypotheses have been proposed regarding the mechanism through which IDO mediates immune tolerance, a precise understanding remains elusive. IDO is an immunoregulatory enzyme that catalyzes a rate-limiting step in tryptophan metabolism via the kynurenine pathway, inhibiting T-cell proliferation and enabling antigens to evade immune surveillance [41]. The enzymatic activity of IDO is essential for immune modulation, exerting biological effects through tryptophan metabolism [27]. Numerous studies have validated the role of IDO in immune regulation and tolerance induction [11]. Investigations in both human and mouse models have examined IDO’s involvement in autoimmune diseases, transplant allograft rejection, and tumor progression [23, 34, 36, 41].

*Trichinella spiralis* has developed mechanisms to establish persistent infections in host skeletal muscles, while evading immune detection [14]. This parasite achieves immune modulation through multiple processes that fine-tune host immune responses. The ES antigen of *T. spiralis* elicits a robust immune response and aids in the protection of infectious larvae. DCs, like APCs, initiate T-cell activation and subsequently regulate immune function. IDO, as an immunomodulatory enzyme, catalyzes the rate-limiting step in tryptophan metabolism and is inhibited by 1-methyl tryptophan (1-MT). Through IDO expression, DCs can induce regulatory T-cells, promoting immune tolerance to autoantigens [13].

The precise mechanism by which *T. spiralis* establishes chronic infection in the host, while evading immune recognition and attack, remains unclear. Further research is required to determine whether and how IDO influences the ability of DCs to induce immunological tolerance to *T. spiralis*. In this study, mouse DCs were cultured *in vitro* and exposed to purified *T. spiralis* ES antigen for varying durations. Flow cytometry was employed to assess the surface expression of CD40, MHC-II, CD80, and CD86 on DCs. Additionally, qRT-PCR was used to analyze the mRNA expression levels of IDO, the proinflammatory cytokines IFN-γ and TNF-α, and the anti-inflammatory cytokine IL-10 at different time points. Western blotting (WB) analysis was performed to evaluate IDO protein expression over time. To confirm the impact of ES antigens on IDO expression in DCs, qRT-PCR was used to measure relative cytokine levels in DCs in which IDO expression was knocked down using small interfering RNA (siRNA620). The results indicated that ES antigens upregulated IDO at both the mRNA and protein levels. Collectively, these findings suggest that IDO expression in DCs may play a significant role in *T. spiralis* infection. This study examined the effect of ES antigens on IDO expression and provided a theoretical basis for further investigation into the molecular mechanisms by which ES antigens influence *T. spiralis* invasion.

## Materials and methods

### Ethics

The feeding and experimental procedures involving mice were conducted in compliance with the Chinese Animal Management Ordinance. The standards for animal experiments were approved by the Animal Management Committee of Northeast Agricultural University and were carried out in accordance with established animal ethics guidelines and approved protocols (Animal Ethics Committee approval number SYXK [Hei] 2016–007).

### Animals and parasites

*Trichinella spiralis* (isolate code: ISS3; original host: domestic pig from Poland) was used as the experimental parasite. A total of 80 Kunming mice (6–8 weeks old, both male and female, randomly selected) were obtained from the Experimental Animal Center of Harbin Medical University, China. The use and care of animals followed the guidelines outlined in the Chinese Animal Management Ordinance.

### Preparation of the excretory secretion antigen

Each mouse was orally infected with 500 larvae. Muscle larvae from infected mice were isolated using pepsin hydrochloride digestion and maintained at 37 °C in 5% CO_2_ in RPMI 1640 medium (HyClone, Logan, UT, USA) supplemented with 1% penicillin/streptomycin (PS) for 24–36 h. ES products were subsequently collected from the cultured muscle larvae, concentrated using polyethylene glycol (PEG2000, 400 g/L), and subjected to dialysis. The ES products were then filtered through a 0.22-μm membrane and stored at −80 °C until use. Protein concentration was determined using the bicinchoninic acid (BCA) assay. A standard curve was generated based on the absorbance of the BCA standard at 562 nm, and the protein content of the sample was calculated assuming a linear relationship. ES antigen composition was analyzed using sodium dodecyl sulfate-polyacrylamide gel electrophoresis (SDS-PAGE).

### Cultivation of dendritic cells

DC2.4 cells were obtained from BeNa Culture Collection Biotechnology Co., Ltd., Langfang, China. Cells were incubated at 37 °C in 5% CO_2_ for 1.5 h. After nonadherent cells were removed, the remaining cells were washed twice with RPMI 1640 medium (HyClone) supplemented with 10% fetal bovine serum (FBS; Sigma-Aldrich, St. Louis, MO, USA). Fresh cultivation medium (RPMI 1640 + 10% FBS + 1% penicillin/streptomycin) was added, and the cells were maintained at 37 °C in 5% CO_2_ for 24 h.

### *In vitro* dendritic cells stimulation

DC2.4 cells were prepared according to previously established methods. Briefly, cells (1 × 10^6^ cells/well) were seeded in 12-well plates and incubated for 24 h. Adherent cells were then divided into four groups and stimulated with different treatments: (1) ES products from *T. spiralis* L1 larvae (final concentration: 15 μg/mL), (2) IFN-γ (final concentration: 1,000 U/mL, positive control), (3) RPMI 1640 (negative control), and (4) blank control. After incubation at 37 °C in 5% CO_2_, the culture medium was removed. The cells were subsequently washed with PBS and stored at −80 °C for RNA extraction.

### Effects of different concentrations of the excretory secretion antigen on the proliferation of dendritic cells

Healthy DCs were seeded in 96-well plates at a density of 1 × 10^6^ cells/well. The culture medium volume was adjusted to 100 μL, and ES antigen at final concentrations of 0, 5, 10, 15, 25, 30, or 40 μg/mL was added to stimulate cells for 24 h. An equal volume of RPMI 1640 was used as a negative control. Following incubation, the supernatant was discarded, and 10 μL of CCK-8 reagent along with 90 μL of culture solution was added to each well in darkness. The cells were incubated at 37 °C for 4 h. Optical density (OD) at 450 nm was measured to assess cell proliferation.

### Time effect of the excretory secretion antigen on indoleamine 2, 3-dioxygenase expression in dendritic cells

The optimal ES antigen concentration was used to investigate the time-dependent effect on IDO expression. Healthy DCs were seeded in six-well plates at a density of 1 × 10^6^ cells/well, with the total volume adjusted to 2 mL. The cells were stimulated with ES antigen for 0, 4, 8, 12, 16, 20, 24, and 32 h. Total RNA was extracted at each time point and reverse-transcribed into cDNA. The expression levels of β-actin and IDO mRNA were analyzed using qRT-PCR.

### Concentration effect of excretory secretion antigen on the expression of indoleamine 2, 3-dioxygenase in dendritic cells

Healthy DCs were seeded in six-well plates at a density of 1 × 10^6^ cells/well, with the culture medium adjusted to a total volume of 2 mL. ES antigen was added at final concentrations of 0, 5, 10, 15, 20, 30, and 40 μg/mL and incubated for 24 h. An equal volume of RPMI 1640 was used as a negative control, while IFN-γ (1,000 U/mL) served as a positive control. Following incubation, total RNA was extracted, and cDNA was synthesized through reverse transcription. The expression levels of β-actin and IDO mRNA were subsequently quantified using qRT-PCR.

### Quantitative real-time polymerase chain reaction

Total RNA was extracted from DCs stimulated for different durations (0, 2, 6, 12, 24, 36, 48, and 72 h) using a Trizol RNA extraction kit (Invitrogen, Vienna, Austria), following the manufacturer’s instructions. cDNA synthesis was performed using a reverse transcription system (Promega, Madison, WI, USA). The primers for PCR were designed using the Primer-BLAST tool from the NCBI website. The primers used were as follows: IDO (accession number, NM_008324), AGCAATCCCCACTGTATCCA, GGTCCACAAAGTCACGCATC, 131 bp; IL-10 (NM_010548), TGGACAACATACTGCTAACCGA, ACCCAGGGAATTCAAATGC, 158 bp; IFN-γ (NM_008337.4), AGTGGCATAGATGTGGAA, CTGTTGCTGAAGAAGGTAG, 200 bp; TNF-α (NM_013693.3), ACGCTCTTCTGTCTACTGA, ACGCTCTTCTGTCTACTGA, 240 bp; β-actin (NM_007393.5), GTTGGAGCAAACATCCCCCA, ACGCGACCATCCTCCTCTTA, 187 bp. Real-time quantitative PCR for IDO, IL-10, IFN-γ, TNF-α, and β-actin was performed in triplicate using the ABI 7500 Real-Time PCR System (Applied Biosystems, Carlsbad, CA, USA) and ChamQTM Universal SYBR qPCR Master Mix (Vazyme Biotech Co., Ltd., Nanjing, China). The qPCR conditions were as follows: initial denaturation at 95 °C for 70 s, followed by 40 cycles of PCR (95 °C for 30 s, 95 °C for 10 s, and 60 °C for 30 s). The melting curve analysis included cycles at 95 °C for 15 s, 60 °C for 1 min, and 95 °C for 15 s. Target gene expression was normalized to β-actin as an internal control. Relative expression levels were determined using the 2^–∆∆Ct^ method.

### Western blotting assay

DCs were stimulated with different antigens for specified durations, washed, and collected for total protein extraction using a protein isolation kit (KeyGEN BioTECH Co., Ltd., Nanjing, China). The extracted proteins were separated via 12% SDS-PAGE and subsequently transferred onto nitrocellulose membranes. To block nonspecific binding, membranes were incubated with 5% (w/v) defatted milk powder dissolved in TBST buffer (0.02% Tween 20, 150 mmol/L NaCl, and 20 mmol/L Tris-HCl, pH 7.6). After five washes with TBST (5 min each), membranes were incubated with a primary mouse IDO monoclonal antibody (1:7,000 dilution) and a mouse GAPDH monoclonal antibody (1:7,000 dilution, reference control) (Peprotech, Rocky Hill, NJ, USA) at 37 °C for 2 h. Following another set of five TBST washes (5 min each), membranes were incubated with horseradish peroxidase (HRP)-labeled goat anti-mouse IgG secondary antibodies at 37 °C for 45 min under continuous agitation. After an additional set of TBST washes, enhanced chemiluminescence (ECL) detection (HaiGene Co., Ltd., Harbin, China) was performed, according to the manufacturer’s instructions. IDO protein expression was quantified using ImageJ software, with GAPDH serving as an internal control for normalization.

### Flow cytometry

Healthy DCs were seeded in six-well plates and cultured in standard RPMI 1640 medium. Cells were either left untreated, stimulated with ES antigen, or treated with lipopolysaccharide (LPS, positive control) for 24 h, with each condition performed in triplicate. Following incubation, cells were washed with PBS, collected, resuspended, and incubated in the dark with fluorescently labeled antibodies for 40 min. After incubation, cells were washed with PBS, centrifuged at 1,000 rpm for 5 min at room temperature, and resuspended for flow cytometric analysis. Flow cytometry was used to assess the expression levels of MHC-II, CD40, CD80, and CD86.

### siRNA interference

Based on the GenBank murine IDO sequence (accession number: NM_008324), three siRNA sequences (siRNA 339, siRNA 620, and siRNA 890) and a control siRNA (Ctrl siRNA) were designed and synthesized by Suzhou Jima Gene Co. Lipofectamine 2000 and siRNA were separately diluted at a 1:40 ratio in serum-free medium and incubated for 5 min. The two solutions were then mixed and allowed to incubate for 20 min to form transfection complexes. DCs were cultured in medium alone (control) or transfected with Ctrl siRNA, siRNA 339, siRNA 620, or siRNA 890 using the prepared transfection reagent. After 6 h of coincubation, cells were stimulated with the ES antigen for 24 h. Subsequently, cells were harvested for qRT-PCR and Western blotting (WB) assays to assess IDO expression levels.

The primers used for these genes were as follows: siRNA620: GCAAUAUUGCUGUUCCCUATT, UAGGGAACAGCAAUAUUGCTT; siRNA890: GCACUGCACGACAUAGCUATT, UAGCUAUGUCGUGCAGUGCTT; siRNA339: GCAGAAUUCCUCCAGGAAATT, UUUCCUGGAGGAAUUCUGCTT; control siRNA: UUCUCCGAACGUGUCACGUTT, ACGUGACACGUUCGGAGAATT; GAPDH: CACUCAAGAUUGUCAGCAATT, UUGCUGACAAUCUUGAGUGAG. The DCs received ES stimulation after transfection and then the relative mRNA expression of IFN-γ, TNF-α, and IL-10 was determined by qRT-PCR analysis.

### Statistical analysis

All data are expressed as the mean ± SD and analyzed statistically using GraphPad Prism 5. Band intensities were quantified with ImageJ software. Group differences were evaluated using one-way analysis of variance (ANOVA). A *p*-value of < 0.05 was considered statistically significant.

## Results

### Effect of the excretory secretion antigen on the proliferation of dendritic cells

**Figure 1:**
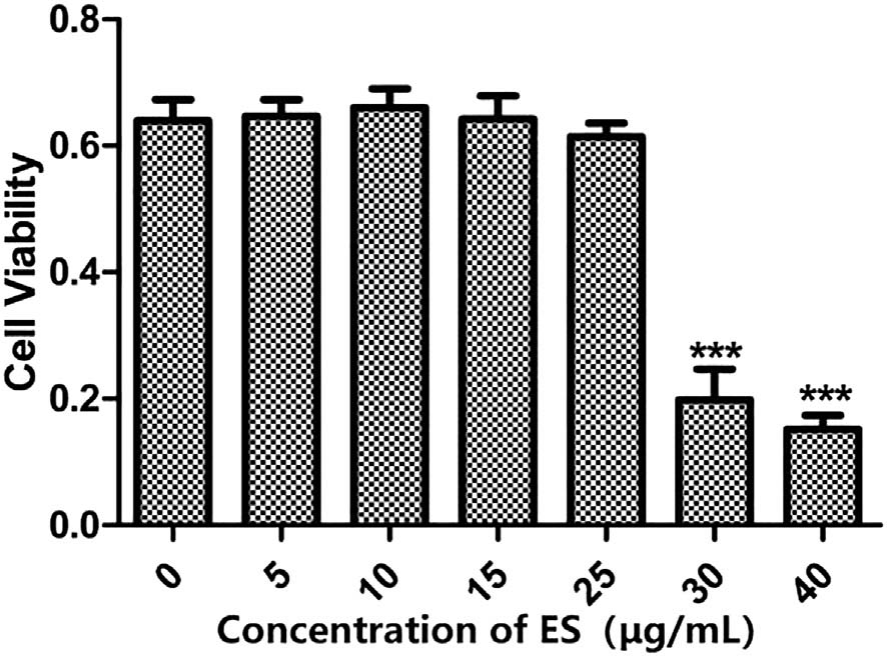
Effect of ES antigen on DC activity. DCs were treated with 0, 5, 10, 15, 25, 30, or 40 μg/mL ES antigen for 24 h. CCK-8 assay was performed to assess cell proliferation activity, and optical density (OD) was measured at 450 nm. ***p < 0.001.

As illustrated in Figure 1, exposure of DCs to the ES antigen for 24 h at a low concentration (0–25 μg/mL) did not influence cell proliferation, with no significant variations observed among different concentrations. However, when the ES antigen concentration exceeded 30 μg/mL, a reduction in cell proliferation activity was noted (*p* < 0.001), indicating cytotoxic effects at higher concentrations. Thus, ES antigen concentrations below 25 μg/mL exhibited no toxic effects on DCs and did not interfere with the experiment.

### Effects of different concentrations of the excretory secretion antigen on the expression of indoleamine 2, 3-dioxygenase in dendritic cells

**Figure 2:**
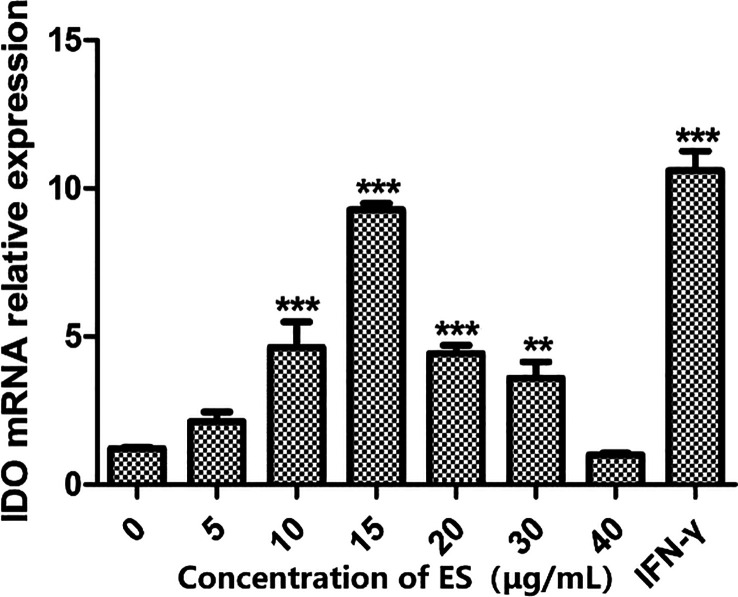
Relative IDO mRNA expression in DCs stimulated with different doses of ES antigen. DCs were treated with 0, 5, 10, 15, 20, 30, or 40 μg/mL ES antigen for 24 h. IFN-γ was used as a positive control. Real-time RT-PCR was conducted to quantify the relative expression of IDO mRNA in DCs. **p < 0.01, ***p < 0.001.

As shown in Figure 2, variations in IDO mRNA expression in DCs were observed in response to different ES antigen concentrations. qRT-PCR assay results indicated that IDO expression was negligible in the absence of ES antigen stimulation. When the ES antigen concentration ranged from 0 to 15 μg/mL, IDO mRNA expression in DCs increased with rising antigen concentrations. However, at concentrations exceeding 20 μg/mL, IDO mRNA expression declined sharply, possibly due to the cytotoxic effects of the ES antigen on cells. Based on these findings, 15 μg/mL was determined to be the optimal concentration for stimulation.

### Time effect of the excretory secretion antigen on indoleamine 2, 3-dioxygenase expression in dendritic cells

**Figure 3:**
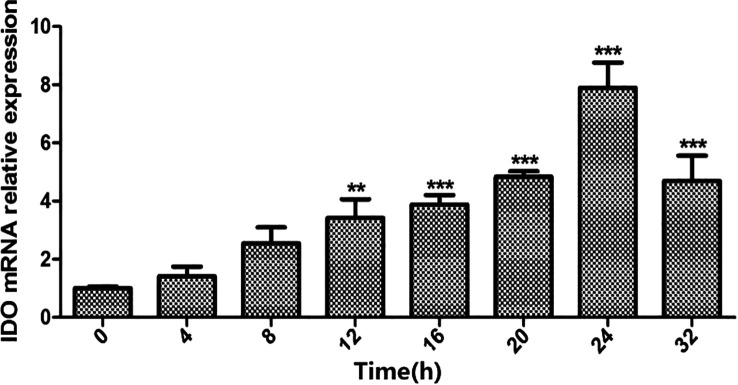
Time-dependent effect of ES antigen on IDO mRNA expression in DCs. DCs were treated with ES antigen at the optimal concentration for 0, 4, 8, 12, 16, 20, 24, or 32 h. Real-time RT-PCR was performed to determine the relative expression of IDO mRNA in DCs. **p < 0.01, ***p < 0.001.

As illustrated in Figures 1 and 2, a 15 μg/mL concentration of ES antigen exhibited no cytotoxic effects on cells and maximized IDO expression in DCs, establishing it as the optimal stimulation concentration. As shown in Figure 3, the relative expression of IDO mRNA in DCs increased within 0–24 h following ES antigen stimulation, followed by a decline after 24 h. At 24 h, IDO mRNA expression was significantly different from that in other groups (*p* < 0.001). The experimental data demonstrated that the same ES antigen concentration that induced the highest IDO secretion by DCs exerted its strongest effects at 24 h. Therefore, under optimal ES antigen concentration, 24 h was identified as the optimal stimulation duration.

### Indoleamine 2, 3-dioxygenase mRNA expression in excretory secretion-stimulated dendritic cells

**Figure 4:**
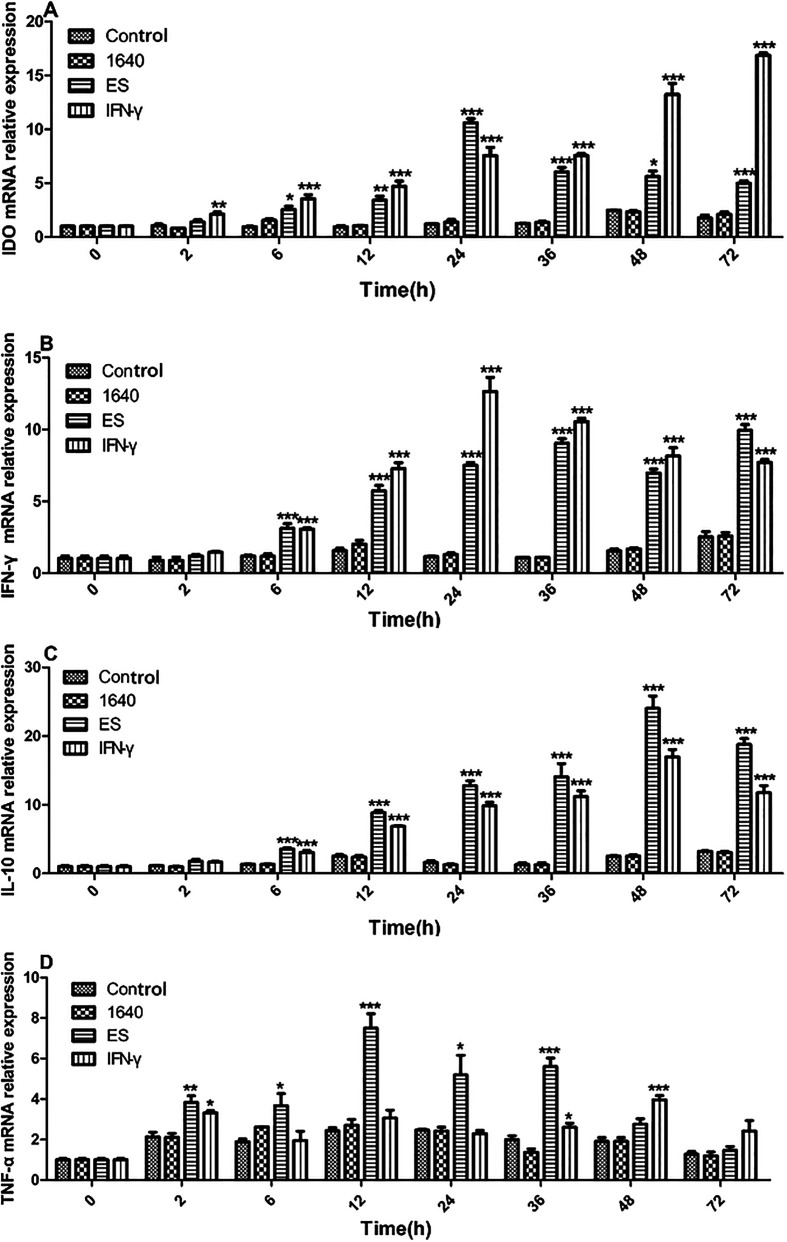
Effects of *T. spiralis* ES products on the expression of IDO and cytokine mRNAs in DC2.4 cells. DCs were treated with 15 μg/mL ES products from *T. spiralis* for 0, 2, 6, 12, 24, 36, 48, or 72 h. IFN-γ was used as a positive control in these experiments. (A–D) Real-time RT-PCR was performed to identify the relative expression of IDO, IFN-γ, IL-10, and TNF-α mRNAs in DCs. *p < 0.05, **p < 0.01, and ***p < 0.001.

To further investigate the mechanisms through which IDO promotes immunologic tolerance in *T. spiralis* and to assess whether the ES antigen upregulates IDO in DCs, the expression levels of IDO, TNF-α, IFN-γ, and IL-10 mRNAs were quantified using qRT-PCR. Cells were cultured for 0, 2, 6, 12, 24, 36, 48, or 72 h under different stimuli. The results indicated a positive correlation among the expression levels of IDO, TNF-α, IFN-γ, and IL-10 mRNAs within 24 h of stimulation, followed by a decline in IDO expression from 36 h to 72 h. Stimulation with IFN-γ or the ES antigen led to significantly higher mRNA expression of IDO (Fig. 4A), IFN-γ (Fig. 4B), IL-10 (Fig. 4C), and TNF-α (Fig. 4D) compared to the blank control. Additionally, IDO was constitutively expressed in the presence of the ES antigen, following a pattern similar to that of IFN-γ. No significant difference was observed between the ES group and the IFN-γ group. In contrast, IDO, TNF-α, IFN-γ, and IL-10 were minimally expressed in the RPMI 1640 group, with no significant difference from the blank control group.

### Upregulation of indoleamine 2, 3-dioxygenase protein expression in dendritic cells

**Figure 5:**
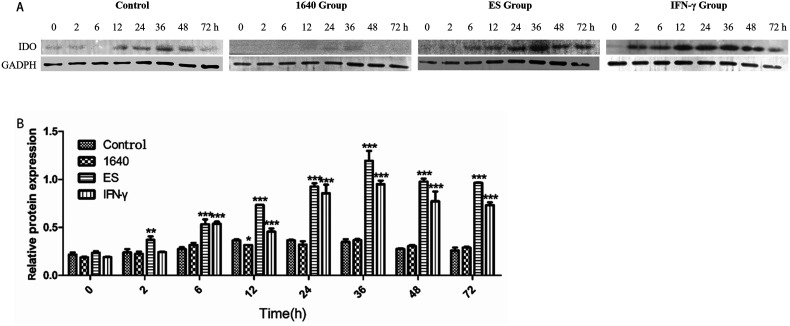
Detection of IDO expression by Western blotting after different DC treatments. (A) Representative bands of the IDO protein in each group were analyzed by WB. (B) The gray value of the IDO protein in different treatment groups was analyzed. *p < 0.05, **p < 0.01, and ***p < 0.001.

WB analysis was conducted to determine whether IDO protein production in DCs aligned with the observed transcriptional upregulation. The results confirmed IDO expression in DCs, with sustained elevation observed from 6 h to 36 h following treatment with IFN-γ and the ES antigen (Fig. 5A). Compared to the blank control group, the grayscale ratio of IDO to GAPDH in DCs stimulated by ES or IFN-γ was higher at 36 h (Fig. 5B). An increase in IDO protein levels was detected after treatment with IFN-γ or ES alone (Fig. 5B).

### Expression of surface marker molecules in excretory secretion-stimulated dendritic cells

**Figure 6:**
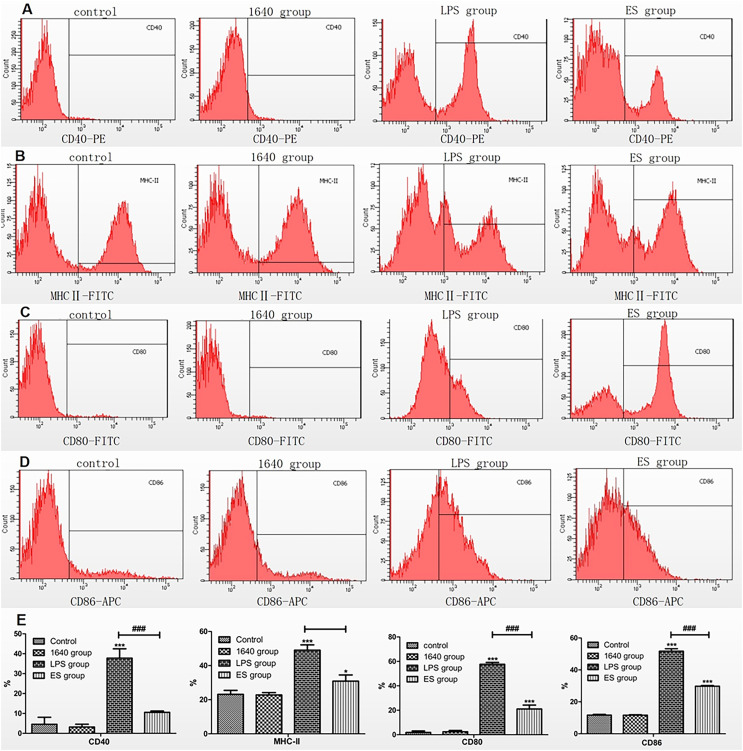
Expression of DC surface marker molecules induced by ES antigen, detected by flow cytometry. DCs were cultured in standard RPMI 1640 medium or stimulated with ES antigen or LPS (positive control) for 24 h. (A–D) Expression levels of CD40, MHC-II, CD80, and CD86 were analyzed by flow cytometry. (E) Ratios of CD40, MHC-II, CD80, and CD86 in different groups. *p < 0.05, **p < 0.01, ***p < 0.001.

The maturation of DCs in each group was assessed by analyzing the expression of surface marker molecules (CD40, CD80, CD86, and MHC-II) using flow cytometry. These markers exhibited low expression in both the blank control group and the 1640 group. In contrast, significant overexpression of surface marker molecules was observed in the LPS group compared to the control group (Figs. 6A–6E). A similar increase in expression levels was detected in the ES antigen group relative to the control group; however, the expression was notably lower than that in the LPS group (Figs. 6A–6E).

### siRNA interference

**Figure 7:**
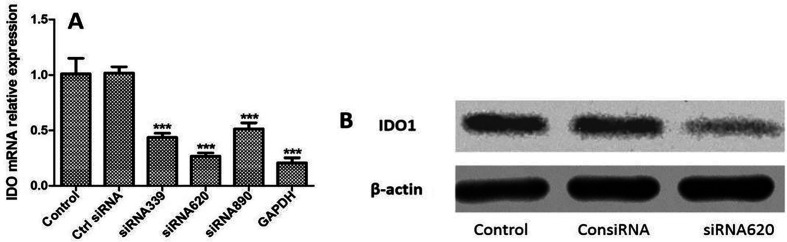
Effects of IDO knockdown on DCs. DCs were cultured in medium alone (control) or transfected with control siRNA (Ctrl siRNA), siRNA 339, siRNA 620, or siRNA 890 for 6 h, followed by stimulation with ES antigen for 24 h. (A) Relative IDO mRNA expression (normalized to GAPDH) was quantified by real-time qRT-PCR. ***p < 0.001. (B) IDO protein levels were analyzed by Western blotting.

**Figure 8:**
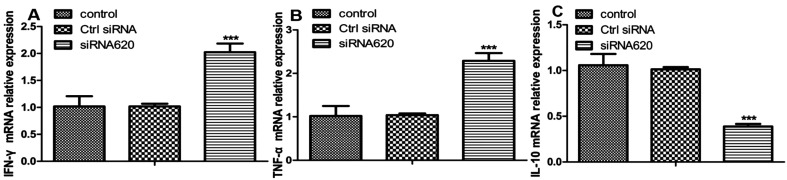
Relative expression of cytokines after siRNA-mediated IDO knockdown. (A) and (B) Relative mRNA expression levels of IFN-γ and TNF-α were quantified by qRT-PCR. The mRNA levels of IFN-γ and TNF-α were significantly higher in the siRNA620 group than in the control and control siRNA groups (***p < 0.001). (C) qRT-PCR analysis of IL-10 mRNA expression. The IL-10 mRNA level was significantly lower in the siRNA620 group than in the control and control siRNA groups (***p < 0.001).

To investigate the role of IDO in immunomodulation, IDO was knocked down in DCs using siRNA. A specific siRNA target for IDO was designed and transfected into DCs for 6 h, followed by stimulation with the ES antigen for 24 h. qRT-PCR analysis revealed that among the three siRNAs tested, siRNA620 achieved a 60% silencing efficiency (Fig. 7A). The protein level in siRNA620-silenced cells was also lower than that in control cells (Fig. 7B). Furthermore, the proinflammatory cytokines IFN-γ (Fig. 8A) and TNF-α (Fig. 8B) exhibited significant increases in mRNA expression, whereas IL-10 mRNA levels showed a contrasting decrease compared to those in the ES antigen group (Fig. 8C).

## Discussion

IDO is a key immunomodulatory enzyme present in various cell types, including DCs and macrophages, and plays a role in regulating immune responses. It facilitates tryptophan degradation while suppressing inflammation and immune activation. Tryptophan depletion contributes to immune resistance by enhancing regulatory T-cell activity and inhibiting effector T-cell function [31]. IDO-mediated tryptophan catabolism has been implicated in autoimmune diseases [6], primary tumors and cancer [21, 46], organ transplantation [9], and placental tolerance [16].

Additionally, IDO is expressed in different cell types, such as DCs and macrophages, and influences various physiological and pathological processes, including anticancer and antimicrobial activities, parasite-induced immune tolerance, and immunoregulation [29]. The helminth *T. spiralis* establishes persistent infections in host skeletal muscles. Depending on the host species, the parasite can survive throughout the host’s lifetime in rodents or persist for months to years in higher mammals and humans, causing chronic parasitic disease [14, 40]. Throughout its life cycle, *T. spiralis*-derived ES products modulate immunity, benefiting both the host and the parasite. These ES products function as antigens that either stimulate prolonged specific immune responses or act as immunomodulators to alter immune reactions to unrelated antigens [14].

Upon *T. spiralis* invasion, the parasite and its ES antigen influence the expression of DC surface markers and cytokine secretion, triggering immune responses that impact host immunity. The ES antigen stimulates immune cells, including macrophages and DCs, and modulates adaptive immunity, altering immune cell function [33]. DCs secrete both inflammatory and anti-inflammatory cytokines, which influence resistance to pathogenic invasion, leading to a shift from a Th1-dominant response to a Th2 or mixed Th1/Th2 immune response [22]. Bai *et al.* [4] reported that stimulation of DCs with the ES antigen of *T. spiralis* led to a significant increase in CD80 and CD86 expression, activating the inflammatory signaling pathway and inducing inflammatory cytokine production. The promotion of inflammatory factor secretion may serve as a key mechanism through which *T. spiralis* evades host immune responses.

When tryptophan is depleted in the T-cell microenvironment, T-cell proliferation is inhibited [28]. Metabolites generated from tryptophan degradation contribute to T-cell apoptosis [37], further impairing immune function. The ES antigen of *T. spiralis* has been shown to stimulate DCs to produce IDO, which indirectly influences T-cell function. In this study, the expression of IDO in DCs following stimulation with the ES antigen of *T. spiralis* and its role in DC maturation *in vitro* were examined. The transcriptional and protein expression levels of IDO in DCs stimulated with the ES antigen were assessed. qRT-PCR results demonstrated an increase in the expression of the anti-inflammatory cytokine IL-10 following stimulation with the ES antigen. The anti-inflammatory effects of IL-10 were primarily observed in the inhibition of activation and cytotoxicity of APCs, including macrophages. The level of expression of the IDO mRNA increased, and the level of expression of the anti-inflammatory factor IL-10 also increased. The IL-10 induced by the antigen presented by DCs is closely associated with the ability of the parasite to evade host immunity. Therefore, the stimulatory effect of the ES antigen plays a crucial role in the evasion of host immunity *in vitro*, self-survival, and growth of *T. spiralis* [15]. IL-10 can also mobilize the expression of IDO in APCs [2, 19].

In this study, elevated IL-10 mRNA expression was observed to enhance the expression of IDO mRNA. An increase in IDO levels in DCs may contribute to the early immune evasion of parasites by reducing T-cell priming and effector T-cell migration due to localized L-tryptophan depletion. The findings indicated that the ES antigen of *T. spiralis* induces IDO expression in DCs, while the sustained high expression of IL-10 maintains IDO activity. This mutual regulation between IDO and IL-10 may support the long-term survival of *T. spiralis* within the host and facilitate immune evasion. The ES antigen of *T. spiralis* was found to suppress T-cell function by decreasing tryptophan concentrations, enabling the parasite to evade host immune responses while promoting the expression of anti-inflammatory factors. Previous studies have demonstrated that IDO plays a critical role in host-pathogen interactions and the immune evasion mechanisms of parasites. Based on these findings, it was hypothesized that increased IDO expression in DCs may be associated with immunomodulatory effects triggered by the ES antigen. Higher IDO expression in DCs was found to reduce T-cell stimulation while suppressing local T-cell responses against the ES antigen of *T. spiralis*. This IDO-mediated mechanism supports *T. spiralis* in evading host immunity, thereby promoting its survival and contributing to chronic parasitic infection.

We also investigated how the ES antigen of *T. spiralis* trigger IDO activation. IFN-γ is the classic hallmark of inflammation that stimulates immunity. Various inflammatory factors, such as TNF-α, IFN-γ, and IFN-α, can induce IDO in DCs [38]. IDO is also induced by IL-10 in certain cell types [27, 43]. During *T. spiralis* infection, significant inflammatory responses occur, leading to the release of various factors, including IFN-γ, which may act as IDO-inducing agents. Recent studies have shown that IFN-γ strongly upregulates IDO and plays a critical role in immune tolerance following allogeneic skin transplantation [48]. Therefore, an increase in IFN-γ may enhance IDO expression, suggesting that IFN-γ could induce IDO to facilitate *T. spiralis* immune evasion. In this study, IL-10 mRNA levels were found to be elevated in DCs following stimulation with the ES antigen of *T. spiralis*. These findings align with those reported by Yu *et al.*, who observed increased IL-10 mRNA expression and higher serum IL-10 levels in *T. spiralis*-infected mice [45]. The expression of IDO promotes the degradation of tryptophan and the accumulation of kynurenine metabolites, which suppress T-cell proliferation and contribute to the induction of regulatory T-cells [5].

The activation of DCs is essential for activating the immune response mechanism. Studies have shown that silencing IDO can increase the ability of DCs to stimulate T-cells *in vitro* [10]. The findings of this study suggest that inhibiting IDO expression in DCs is essential for determining whether IDO contributes to the immune evasion induced by the *T. spiralis* ES antigen. An effective siRNA targeting IDO was used to interfere with DC function, and a silencing effect was observed at a specific concentration. Prior research has shown that IDO-silenced DCs improve the efficacy of DC immunotherapy by enhancing tumor-specific immune responses [7]. IDO expression is linked to T-cell response suppression, as activated T-cells are highly sensitive to local tryptophan depletion mediated by IDO. Furthermore, tryptophan degradation products contribute to T-cell apoptosis [11, 39]. Silencing IDO promotes tumor antigen-specific T-cell proliferation, enhances tumor cell lysis, and decreases the population of CD4^+^ CD25^+^ Foxp3^+^ regulatory T-cells [8]. In a Lewis lung cancer model, IDO silencing significantly reduced tumor growth and delayed tumor formation [32]. Another study [47] reported that patients with metastatic melanoma exhibited T-cell responses to tumor antigens after receiving an IDO-silenced DC vaccine cotransfected with mRNA, leading to a significant reduction in lung, liver, and skin metastases. These findings indicate that IDO silencing provides clinical benefits by enhancing immune responses against tumors and potentially mitigating immune evasion by parasites such as *T. spiralis*.

## Conclusion

This study demonstrated that the ES antigen increased the expression of IDO mRNA and protein in DCs. Additionally, IDO activation by IFN-γ led to upregulation of TNF-α, IFN-γ, and IL-10. The findings indirectly suggested that the sustained increase in IDO expression is associated with the elevated expression of IFN-γ. These results confirmed the interactions among the ES antigen, DCs, and IDO, highlighting the potential immunomodulatory role of the IDO protein. The study further indicated that IDO, as a molecule associated with immune tolerance, plays a role in the establishment of chronic *T. spiralis* infection in the host. These findings provide deeper insights into the molecular pathways regulating IDO activation, which may contribute to the development of novel therapeutic strategies against *T. spiralis* infection.
